# Optimal pharmacological therapy in ST-elevation myocardial infarction—a review: A review of antithrombotic therapies in STEMI

**DOI:** 10.1007/s12471-018-1112-6

**Published:** 2018-04-23

**Authors:** R. S. Hermanides, S. Kilic, A. W. J. van ’t Hof

**Affiliations:** 1 Isala Heart Centre, Zwolle, The Netherlands; 2 Department of Cardiology, Maastricht UMC, Maastricht, The Netherlands; 3 Department of Cardiology, Zuyderland Medical Centre (Heerlen location), Heerlen, The Netherlands

**Keywords:** STEMI, Antithrombotic therapy

## Abstract

Antithrombotic therapy is an essential component in the optimisation of clinical outcomes in patients with ST-elevation myocardial infarction (STEMI) undergoing primary percutaneous coronary intervention. There are currently several intravenous anticoagulant drugs available for primary percutaneous coronary intervention. Dual antiplatelet therapy comprising aspirin and P2Y12 inhibitor represents the cornerstone treatment for STEMI. However, these effective treatment strategies may be associated with bleeding complications. Compared with clopidogrel, prasugrel and ticagrelor are more potent and predictable, which translates into better clinical outcomes. Therefore, these agents are the first-line treatment in primary percutaneous coronary intervention. However, patients can still experience adverse ischaemic events, which might be in part attributed to alternative pathways triggering thrombosis. In this review, we provide a critical and updated review of currently available antithrombotic therapies used in patients with STEMI undergoing primary PCI. Finding a balance that minimises both thrombotic and bleeding risk is difficult, but crucial. Further randomised trials for this optimal balance are needed.

## Introduction

**Fig. 1:**
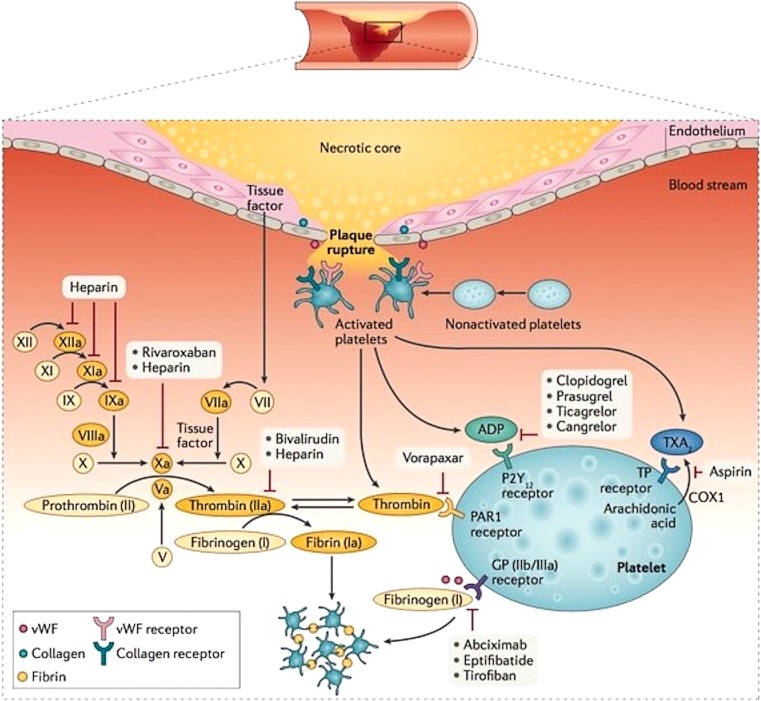
Mechanism of thrombus formation during STEMI, and targets of available antithrombotic agents. After plaque rupture, a complex mechanism of thrombus formation is mediated. *COX* cyclooxygenase, *TP* thromboxane prostanoid,* STEMI* ST-elevation myocardial infarction

Acute ST-elevation myocardial infarction (STEMI) is a major cause of mortality worldwide. The rapid restoration of blood flow in the occluded culprit coronary artery with primary percutaneous coronary intervention (PCI) will prevent heart failure, preserves ventricular function and reduces mortality [1–4]. The cause of STEMI is erosion or rupture of an atherosclerotic plaque with subsequent platelet adherence, activation, aggregation, and activation of the clotting cascade and downstream myocardial ischaemia and necrosis after complete coronary artery occlusion [5]. The main elements involved in this process are platelet and coagulation factors (Fig. 1). It is very important to find a balance in pharmacological management of STEMI that will minimise thrombotic risk and bleeding risk. In this article, we review currently available antithrombotic therapies that can be used in patients with STEMI who are undergoing primary PCI.

**Figure:**
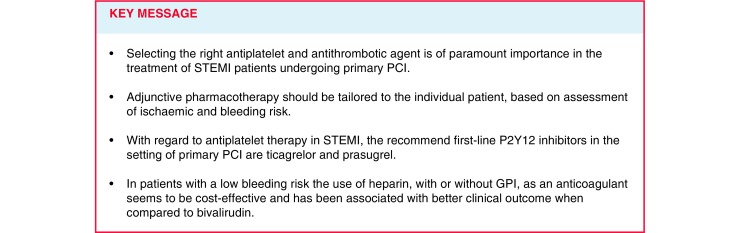

## Antiplatelet and anticoagulant medications for STEMI

### *Classification of anticoagulants (*Tab. 1*)*

1. Unfractionated heparin
2. Low-molecular-weight heparin
3. Bivalirudin
4. Thrombin receptor antagonist protease-activated receptor
  vorapaxar
5. Factor Xa inhibitor
  rivaroxaban

**Table 1:** Pharmacological properties of anticoagulants in STEMI

	UFH	Enoxaparin	Bivalirudin
Administration route	Intravenous	Intravenous, subcutaneous	Intravenous
Factor Xa:IIa inhibition	1:1	3–4:1	Only IIa
Action independent of antithrombin	No	No	Yes
Nonspecific binding	Yes	Partial	No
Variable PK/PD measures	Yes	Yes (<unfractionated heparin)	No
Inhibits fibrin-bound thrombin	No	No	Yes
Effect on platelets	Activation	Activation	Inhibition
Half-life	~60 min	90–120 min	25 min
Risk of HITT	Yes	Yes (<unfractionated heparin)	No
Dose in PPCI	70–100 U/kg bolus without GPIs; 50–70 U/kg bolus with GPIs	0.5 mg/kg intravenous bolus	0.75 mg/kg intravenous bolus; 1.75 mg/kg/h infusion
Reversal agent	Protamine sulfate	No	No

*STEMI* ST-elevation myocardial infarction, *HITT* heparin induced thrombocytopenia and thrombosis, *PPCI* primary percutaneous coronary intervention, *GPIs* glycoprotein IIb/IIIa inhibitors, *PK* pharmacokinetic, *PD* pharmacodynamics *UFH* unfractionated heparin

### Classification of antiplatelet agents

1. Aspirin
2. P2Y12 receptor inhibitors
  clopidogrel, prasugrel (irreversible inhibitors)
  cangrelor and ticagrelor (reversible inhibitors)
3. Glycoprotein IIb/IIIa inhibitors
  tirofiban, abciximab, eptifibatide

### Anticoagulant therapy before and during primary PCI

#### Unfractionated heparin

Based on several small randomised trials, anticoagulation with unfractionated heparin became an accepted and important therapy for STEMI, especially before and during the PCI procedure [6]. The major anticoagulant effect of unfractionated heparin is based on inactivation of thrombin and activated factor X (factor Xa) reacts by binding to antithrombin III, an endogenous inhibitor of factor Xa and thrombin IIa. This binding induces a conformational change in antithrombin 3, which markedly accelerates its ability to inactivate these factors. The recommended peri-procedural dosing in patients who also receive a glycoprotein IIb/IIIa inhibitor is 50 to 70 U/kg (target activated clotting time [ACT] > 200 s); in patients not receiving a GP IIb/IIIa inhibitor, the recommended peri-procedural dosing is 60 to 100 U/kg (target ACT, 250 to 350 s) [3, 4]. Currently, unfractionated heparin has a Class I indication (level of evidence (LOE):C) for anticoagulation during primary PCI in the new European Society of Cardiology (ESC) guideline [3]. A major limitation of unfractionated heparin is the increased risk of heparin-induced thrombocytopenia, a serious, potentially lethal, and immunologically mediated adverse reaction.

#### Low-molecular-weight heparin

Low-molecular-weight heparins seem to offer a better clinical efficacy in STEMI when administered intravenously as compared with unfractionated heparin. In the ATOLL study [7], 900 STEMI patients were randomly assigned to receive an intravenous bolus of 0.5 mg/kg enoxaparin or unfractionated heparin before primary PCI. Comparison of enoxaparin versus unfractionated heparin in the ATOLL study showed that enoxaparin was associated with a significant reduction in the secondary endpoint (a composite of death, recurrent acute coronary syndrome or urgent revascularisation) and a significant reduction in individual endpoints, including mortality, major bleeding, and urgent revascularisation. It is recommended to use enoxaparin, preferentially via intravenous route, 0.5 mg/kg. Based on a potential benefit in the secondary endpoint, the new ESC guidelines give a Class IIa (LOE: A) indication for enoxaparin in this setting [3, 4].

The OASIS-6 trial, which evaluated fondaparinux (factor X inhibitor) use in STEMI patients, received a lot of criticism because of the heterogeneity in the medical and invasive treatment of the enrolled patients and showed a high rate of catheter thrombosis and coronary complications [8]. Fondaparinux is not recommended in the ESC guidelines as background anticoagulant for primary PCI (Class III; LOE: B).

#### Bivalirudin

Bivalirudin is a direct inhibitor of soluble and clot-bound thrombin [9]. It has a rapid onset of action and a half-life of 25 min and is therefore given as an intravenous infusion. The results of several bivalirudin studies (HEAT-PPCI, MATRIX, BRAVE-4, EUROMAX, BRIGHT) have led to discussions about its added value (Tab. 2). Recent meta-analyses have demonstrated no mortality benefit but fewer bleeding complications in bivalirudin users [9–14]. The EUROMAX trial is a randomised clinical trial comparing bivalirudin versus heparin plus optional glycoprotein IIb/IIIa inhibitors in patients undergoing primary PCI for STEMI. The 1‑year mortality outcomes showed that the total number of deaths was identical in both study arms (59 events in each) with no appreciable differences in the two treatment arms between 30 days and 1 year [15], despite a bivalirudin-associated reduction in the occurrence of the study’s primary endpoint which was death or major bleeding at 30 days. The most recent publication, the VALIDATE-SWEDEHEART, a registry-based, randomised, controlled trial that compared bivalirudin with heparin monotherapy in patients with STEMI or non-ST-elevation myocardial infarction (NSTEMI) who underwent PCI (predominantly radial PCI) and received treatment with high-intensity platelet inhibitors, did not find a difference with respect to the rate of death, repeat myocardial infarction, or major bleeding events during 180 days of follow-up [16]. Bivalirudin, with or without previous heparin therapy, has a Class IIa (LOE: A) indication in the new 2017 ESC STEMI guidelines, that also specify that at PCI the recommended dosing is an initial bolus of 0.75 mg/kg and an infusion of 1.75 mg/kg/hr during PCI. Following PCI, an infusion of 0.25 mg/kg/hr can be continued if clinically appropriate [3, 4].

**Table 2:** Major trials of bivalirudin versus UFH in STEMI

	HORIZONS-AMI	EUROMAX	HEAT-PPCI	BRIGHT	MATRIX-STEMI
*Trial design*					
Patient population	3,602 STEMI undergoing PPCI	2,218 STEMI transported for PPCI	1,812 STEMI undergoing PPCI	2,194 acute MI undergoing emergency PCI (87.7% STEMI)	4,010 STEMI undergoing PPCI
Type of heparin	UFH	UFH or enoxaparin	UFH	UFH	UFH
Heparin dose	60 U/kg with subsequent boluses targeted to ACT of 200–250 s	UFH: 100 U/kg without GPI or 60 U/kg with GPI. Enoxaparin: 0.5 mg/kg	70 U/kg	100 U/kg in UFH-only group; 60 U/kg in heparin plus tirofiban group	70–100 U/kg without GPI or 50–70 U/kg with GPI
GPI use in heparin group	Routine (97.7% of patients)	Routine or bailout (69% of patients)	Bailout (14% of patients)	Bailout (5.6% of patients) in UFH-only group; routine (100%) in UFH plus tirofiban group	Routine or bailout (25.9% of patients)
GPI use in bivalirudin group	Bailout (7.5% of patients)	Bailout (11.5% of patients)	Bailout (14% of patients)	Bailout (4.4% of patients)	Bailout (4.6% of patients)
Post-PCI bivalirudin infusion	None (but could be continued at low doses if clinically indicated)	4 h after PCI at 0.25 mg/kg/h; continuation of PCI dose was also permitted (22.5%)	None	30 min–4 h after PCI (mean 180 min) at PCI dose	Patients randomised 1:1 to receive or not receive post-PCI infusion (full dose for up to 4 h or reduced dose of 0.25 mg/kg/h for ≥6 h)
P2Y12 receptor inhibitor	Clopidogrel 300–600 mg	Clopidogrel (50%), prasugrel (30%), or ticagrelor (20%)	Clopidogrel (11%), prasugrel (27%), or ticagrelor (62%)	Clopidogrel 300–600 mg	Clopidogrel (29%), prasugrel (31%), or ticagrelor (30%)
Radial access	No	47% of patients	81% of patients	78% of patients	50% (randomised 1:1 to radial versus femoral)
Primary end point	NACE (major bleeding or MACE [death, reinfarction, TVR for ischaemia, and stroke]) at 30 days; non-CABG-related major bleeding at 30 days	Death or non-CABG-related major bleeding at 30 days	Efficacy: MACE (all-cause death, CVA, reinfarction, or additional unplanned TLR) at 28 days. Safety: major bleeding at 28 days	NACE (MACE [all-cause death, reinfarction, ischaemia-driven TVR, or stroke] or any bleeding) at 30 days	MACE (death, MI, or stroke) at 30 days; NACE (MACE or major bleeding) at 30 days
Bleeding definition	Protocol-defined	Protocol-defined	BARC type 3–5	BARC	BARC type 3 or 5
*Study results*					
Primary end point(s)	NACE: bivalirudin 9.2% vs. UFH 12.1% (*P* = 0.005); non-CABG-related major bleeding: bivalirudin 4.9% vs. UFH 8.3% (*P* < 0.001)	Bivalirudin 5.1% vs. UFH 8.5% (*P* = 0.001)	MACE: bivalirudin 8.7% vs. UFH 5.7% (*P* = 0.02); major bleeding: bivalirudin 3.5% vs. UFH 3.1% (*P* = 0.59)	Bivalirudin 8.8% vs. heparin 13.2% vs. UFH plus tirofiban 17.0% (*P* < 0.001)	MACE: bivalirudin 5.9% vs. UFH 6.5% (*P* = 0.43); NACE: bivalirudin 7.0% vs. UFH 8.2% (*P* = 0.13)
MACE	Bivalirudin 5.4% vs. UFH 5.5% (*P* = 0.95)	Bivalirudin 6.0% vs. UFH 5.5% (*P* = 0.64)	See primary end point	Bivalirudin 5.0% vs. UFH 5.8% vs. UFH plus tirofiban 4.9% (*P* = 0.83)	See primary end point
Major bleeding	See primary end point	Bivalirudin 2.6% vs. UFH 6.0% (*P* < 0.001)	See primary end point	BARC type 3–5: bivalirudin 0.5% vs. UFH 1.5% vs. UFH plus tirofiban 2.1% (*P* = NA)	Bivalirudin 1.7% vs. UFH 2.8% (*P* = 0.019)
Acute stent thrombosis (≤24 h)	Bivalirudin 1.3% vs. UFH 0.3% (*P* < 0.001)	Bivalirudin 1.1% vs. UFH 0.2% (*P* = 0.007)	Bivalirudin 2.9% vs. UFH 0.9% (*P* = 0.007)	Bivalirudin 0.3% vs. UFH 0.3% vs. UFH plus tirofiban 0.3% (*P* = NA)	Bivalirudin 0.9% vs. UFH 0.5% (*P* = 0.10)

*STEMI* ST-elevation myocardial infarction, *UFH* unfractionated heparin, *CABG* coronary artery bypass grafting,* GPI* glycoprotein IIb/IIIa inhibitors, *PPCI* primary percutaneous coronary intervention, *MACE* major adverse cardiac events, *NACE* net adverse clinical events, *MI* myocardial infarction, *BARC* bleeding academic research consortium, *NA* not available, *TVR* target vessel revascularisation, *TLR* target lesion revascularisation

### Antiplatelet therapy before and during primary PCI

#### Aspirin

Aspirin irreversibly blocks both cyclo-oxygenase 1 (COX-1) and COX-2 and inhibits the production of thromboxane A2 [17]. Thromboxane A2 stimulates further platelet activation and aggregation, which is produced by activated platelets. The large ISIS-2 trial showed that use of aspirin was associated with decreased rates of re-infarctions and non-fatal strokes at mid-term follow-up, with the highest benefit seen in patients undergoing PCI [18].

Given its established benefits in secondary prevention [19], aspirin should be used indefinitely in all patients with STEMI. The dosage of aspirin is topic of discussion. In respect of the first few days of treatment, the CURRENT-OASIS 7 trial [20] failed to demonstrate a difference in hard clinical outcomes when comparing low doses (75–100 mg/day) or relatively high doses of 300–325 mg/day. There were, however, fewer gastro-intestinal bleeds with the lower doses. The ESC recommends administering an initial loading dose of 150–500 mg of oral or intravenous acetylsalicylic acid, unless contraindicated, followed by a life-long maintenance dose of 75–100 mg daily (Class I; LOE: B) (Tab. 3; [3]). Patients who are truly intolerant to aspirin may instead receive clopidogrel (75 mg/day) as long-term secondary prevention [21].

**Table 3:** Pharmacological properties of oral antithrombotic therapy in/after STEMI

	Aspirin	Clopidogrel	Prasugrel	Ticagrelor	Vorapaxar	Rivaroxaban
Target	COX1	P2Y12 receptor	P2Y12 receptor	P2Y12 receptor	PAR1	Factor Xa
Type of blockade	Irreversible	Irreversible	Irreversible	Reversible	Reversible	Reversible
Dose	150–325 mg LD; 81–100 mg once-daily MD	600 mg LD; 75 mg once-daily MD	60 mg LD; 10 mg once-daily MD	180 mg LD; 90 mg twice-daily MD	2.08 mg once-daily MD	2.5 mg twice-daily MD
Prodrug	No	Yes	Yes	No	No	No
Onset of action	60 min	2–8 h	30 min–4 h	30 min–4 h	>12 h||	2–4 h
Offset of action	7–10 days	7–10 days	7–10 days	3–5 days	4–8 weeks	12 h
Drug interactions	NSAIDs	CYP2C19 inhibitors	No	CYP3A inhibitors or inducers, drugs using P‑glycoprotein transporter	CYP3A inhibitors or inducers, drugs using P‑glycoprotein transporter	CYP3A4 inhibitors or inducers, P‑glycoprotein transporter inhibitors
Timing of administration	Immediately after presentation	At presentation or at time of primary PCI	At presentation or at time of primary PCI	At presentation or at time of primary PCI	After stabilisation	After stabilisation (>24 h after admission)
Contraindications	Hypersensitivity	Hypersensitivity, active pathological bleeding	Prior CVA, high risk of bleeding, hypersensitivity	Prior ICH, high risk of bleeding, severe hepatic dysfunction, hypersensitivity	Prior ICH or CVA, high risk of bleeding, hypersensitivity	Prior CVA, CrCl <15 ml/min, high bleeding risk, severe hepatic dysfunction, treatment with other anticoagulant, hypersensitivity

*STEMI* ST-elevation myocardial infarction, *COX1* cyclo-oxygenase-1, *PAR-1* protease-activated receptor-1, *LD* loading dose, *MD* maintenance dose, *NSAIDs* non-steroidal anti-inflammatory drugs, *PCI* percutaneous coronary intervention, *CVA* cerebrovascular accident, *ICH* intracerebral haemorrhage, *CrCl* creatinine clearance

#### P2Y12 receptor inhibitors

##### Clopidogrel

The second-generation thienopyridine clopidogrel is the most widely used P2Y12 receptor inhibitor [22]. The clinical use of clopidogrel in acute coronary syndrome has been investigated in the large CURE trial, which evidenced a significant reduction in a composite of cardiovascular death, recurrent acute myocardial infarction or stroke when a 300 mg loading dose followed by a 75 mg daily dose of the drug was added to aspirin versus aspirin alone (9.3% versus 11.4%; *p* < 0.001). Furthermore, the CURRENT-OASIS 7 trial showed benefit of a 600 mg loading dose instead of a 300 mg loading dose [20]. However, there is growing concern about response variability in patients with clopidogrel use [23]. Genetic testing before starting clopidogrel therapy, in high-risk STEMI patients, and platelet function testing, in those who suffer adverse events, may facilitate the monitoring of clopidogrel treatment, and is currently being investigated [24]. The current recommendations by the ESC state a loading dose of 600 mg of clopidogrel followed by a maintenance dose of 75 mg daily (or 150 mg until day 8) only when prasugrel or ticagrelor are either not available or contraindicated (Class I; LOE: A) (Tab. 3; [3]).

##### Prasugrel

Prasugrel is an oral, third-generation thienopyridine. Like clopidogrel, it is a prodrug, and thus needs to be metabolised via cytochrome P450 in the liver to produce an active metabolite. The PRINCIPLE-TIMI trial showed that a 60 mg loading dose and 10 mg maintenance dose of prasugrel achieved superior results in terms of platelet inhibition compared with a 600 mg loading dose and 150 mg maintenance dose of clopidogrel [25]. The TRITON-TIMI 38 study compared a 60 mg loading dose followed by 10 mg daily maintenance dose of prasugrel with a 300 mg loading dose and 75 mg daily maintenance dose of clopidogrel, with the loading dose administered after coronary angiography [26]. The trial demonstrated a significant reduction of the composite endpoint of cardiovascular death, non-fatal acute myocardial infarction and non-fatal stroke in the prasugrel group (9.9% versus 12.1%; *p* < 0.001), with early survival advantages after only 3 days persisting at a mean follow-up of 14.5 months. The ESC recommends a 60 mg loading dose and 10 mg daily maintenance dose of prasugrel in patients undergoing PCI (Class I; LOE: A) after visualisation of the coronary arteries (Tab. 3; [3, 4]). Of note, prasugrel is contraindicated in patients with prior stroke/transient ischaemic attack and its use is generally not recommended in patients aged ≥75 years or in patients with lower body weight (<60 kg).

##### Ticagrelor

Ticagrelor is a class of adenosine diphosphate (ADP) blockers, triazolopyrimidines, which act as ADP analogues directly binding to P2Y12 causing allosteric reversible blockage of the receptor. The PLATO trial [27], comparing a standard 300–600 mg loading dose and 75 mg daily maintenance dose of clopidogrel with a 180 mg loading dose and 90 mg twice daily maintenance dose of ticagrelor, showed a significant reduction in the composite primary endpoint of cardiovascular deaths, acute myocardial infarction and non-fatal strokes (9.8% ticagrelor group versus 11.7% clopidogrel group; *p* < 0.001), mainly driven by a reduction of deaths (4.0% versus 5.1%) and acute myocardial infarction (5.8% versus 6.9%). Treatment with ticagrelor was associated with significantly higher rates of bleeding not related to coronary artery bypass graft (4.5% versus 3.8%; *p* = 0.03) or spontaneous bleeding (3.1% versus 2.3%; *p* = 0.01). As expected, dyspnoea was more frequent in patients treated with ticagrelor (13.8% versus 7.8%; *p* < 0.001), even if this was not a significant cause of treatment discontinuation. These PLATO findings were confirmed in the real-world SWEDEHEART registry, however, in SWEDEHEART, ticagrelor was preferentially used in patients with a low bleeding risk and death, and patients on ticagrelor were significantly more often assessed with coronary angiography and treated with PCI [28, 29].

Pre-hospital treatment with P2Y12 receptor inhibitors in STEMI was tested in the ATLANTIC trial [30], defined as administration of loading dose before coronary angiogram, to provide stronger platelet inhibition. This trial showed a significant reduction in the rate of acute stent thrombosis with no difference in major bleeding. Pre-PCI markers of coronary reperfusion did not improve with pre-hospital use of ticagrelor, whereas post-PCI reperfusion did [31]. Of note, the ESC recommends that patients undergoing primary PCI receive a combination of dual antiplatelet therapy as early as possible before angiography.

The new ESC guidelines recommend a ticagrelor 180 mg loading dose followed by 90 mg twice daily in all intermediate to high-risk acute coronary syndrome patients (Class I; LOE: A) (Tab. 3 and 4; [3]).

**Table 4:** Pharmacology of glycoprotein IIb/IIIa inhibitors

	Abciximab	Eptifibatide	Tirofiban
Trade name	ReoPro	Integrilin	Aggrastat
Molecule	Fragment antigen binding (Fab) 7E3	Synthetic peptide	Non-peptide mimetic
Molecular weight (Da)	~50,000	~800	~500
Stoichiometry (drug to glycoprotein IIb/IIIa)	~1.5:1	>>100:1	>>100:1
Binding	Non-competitive	Competitive	Competitive
Half-life (h)	Plasma: 10–15	Plasma: 2.0–2.5	Plasma: 2.0–2.5
	Biologic: 12–24	Biologic = plasma	Biologic = plasma
PCI dosing	Bolus: 250 μg/kg	Bolus: 180 μg/kg* plus 180 μg/kg (after 10 min)	Bolus: 25 μg/kg
	Infusion: 0.125 μg/kg/min (12 h)	Infusion: 2 μg/kg/min (12–24 h)‡	Infusion: 0.15 μg/kg/min (up to 18 h)
Renal adjustment	No	Bolus: 180 μg/kg	Bolus: 25 μg/kg
		Infusion: 1 μg/kg/min (12–24 h)	Infusion: 0.075 μg/kg/min (up to 18 h)

*PCI* percutaneous coronary intervention, *Da* dalton

#### Switching to clopidogrel after ticagrelor pre-treatment

**Fig. 2:**
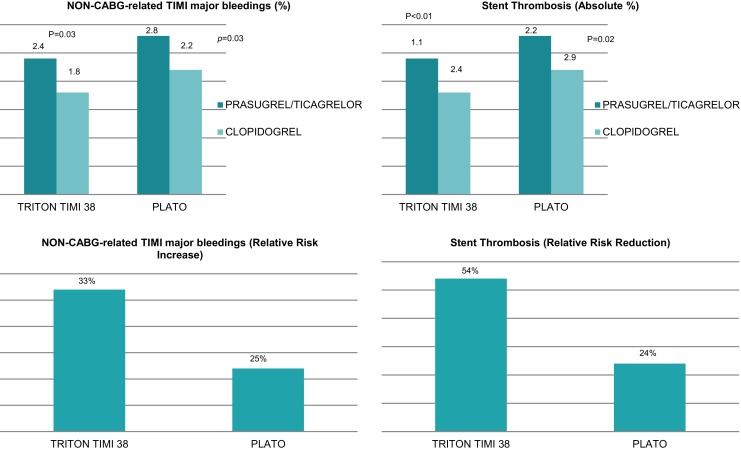
Antiplatelet therapy outcomes in major trials. *CABG* coronary artery bypass grafting, *TIMI* thrombolysis in myocardial infarction

Based on PLATO data, the more potent P2Y12 inhibitor ticagrelor is preferred over clopidogrel in the STEMI setting (Fig. 2). However, a recently published Dutch observational study (40% STEMI patients) demonstrated (based on a propensity score-adjusted multivariate analysis) that in the era of current second-generation drug-eluting stents treatment with ticagrelor compared with clopidogrel was an independent predictor of net adverse clinical and cerebral events (NACCE) and major bleeding [32]. Furthermore, in the TOPIC study patients with acute coronary syndrome treated with PCI were randomised after one month of dual antiplatelet therapy with ticagrelor, to continued treatment with ticagrelor until 12 months, or to switching to clopidogrel. The main outcome consisted of a net clinical benefit for the switched group, primarily driven by a significantly higher bleeding risk in patients on a continued potent P2Y12 inhibitor [33].

#### Prasugrel versus ticagrelor

There are no studies in which the impact of ticagrelor versus prasugrel on outcome in patients with STEMI was tested directly. An adjusted indirect comparison meta-analysis [34] of prasugrel versus ticagrelor in patients with acute coronary syndrome has shown that both drugs are superior to clopidogrel. Head-to-head comparison suggests similar efficacy and safety of prasugrel and ticagrelor, but prasugrel appears more protective against stent thrombosis, especially in the early phase of post-stent implantation.

#### Cangrelor

Cangrelor is an intravenous direct-acting P2Y12 blocker with an almost immediate antiplatelet effect, a plasmatic half-life of 3–5 min and rapid restoration of platelet function just 1 h after infusion cessation. The large CHAMPION series, including STEMI patients in CHAMPION PCI and CHAMPION PHOENIX, showed by pooled analysis [35] a significant reduction in stent thrombosis at 30 days (0.9% versus 1.3%, *p* = 0.0027). We certainly need future large trials to evaluate the safety and benefits of cangrelor in primary therapy, especially as upstream therapy with the aim of abortion of infarction. The Food and Drug Administration (FDA) and the European Medical Agency (EMA) have approved the use of cangrelor (30 μg/kg bolus plus 4 μg/kg/min infusion initiated before PCI and continued for ≥2 h or for the duration of PCI, whichever is longer) for clinical use by as adjunct therapy to PCI for reducing the risk of peri-procedural myocardial infarction, repeat coronary revascularisation, and stent thrombosis in patients who have not been treated with an oral P2Y12 receptor antagonist and are not being given a glycoprotein IIb/IIIa inhibitor. Cangrelor may be considered in patients who have not received P2Y12 receptor inhibitors according to current ESC guidelines (Class IIb; LOE: A) [3].

#### Glycoprotein IIb/IIIa inhibitors (GPIs)

GPIs can be classified into two groups: small (eptifibatide, tirofiban) and non-small (abciximab) molecules, which are characterised by different pharmacological properties (Tab. 4). They target the final pathway of platelet aggregation, competing with von Willebrand factor and fibrinogen for glycoprotein IIb/IIIa receptor binding and provide fast and potent antiplatelet effects. There are several randomised trials examining abciximab versus placebo without potent P2Y12 inhibitors in STEMI (RAPPORT, ADMIRAL, ISAR-2, CADILLAC, and ACE trial). In a meta-analysis of these trials, abciximab was associated with significant reductions in mortality at 30 days and 6 to 12 months and in re-infarction at 30 days [36]. In addition, there was no increase in bleeding. In the past few years several other GPI trials (ON-TIME 2 trial, HORIZONS-AMI trial, FINESSE) were conducted, with conflicting results. The last two GPI trials, EUROMAX and MATRIX, investigated GPI and heparin versus bivalirudin and were discussed in the bivalirudin section.

Use of GPI has waned by the introduction of new P2Y12 inhibitors. However, recent data have clearly shown a delayed onset of action of both prasugrel and ticagrelor [37].

In our opinion GPI can be recommended as early as possible (upstream strategy) among high-risk STEMI patients, such as those with advanced Killip class or anterior myocardial infarction, and those presenting within the first three hours after symptom onset [38, 39].

This was also shown in several pre-specified sub-analyses in the ON-TIME 2 trial.

The ON-TIME 2 randomised trial showed that when tirofiban is administered in the pre-hospital setting as double bolus in association with 600 mg clopidogrel, aspirin, and heparin this results in beneficial effects in terms of an average reduction of the ST-segment 1 h after primary PCI and better clinical outcome at 1 year compared with placebo [40, 41].

Moreover, pre-hospital administration of tirofiban reduces initial thrombus burden, improves initial patency of the infarct-related vessel before primary PCI, and suggests that pre-hospital use is superior to provisional use as a bailout strategy [42–44].

Based on the INFUSE-AMI trial [45] (reduction in infarct size at day 30 on magnetic resonance imaging) and AIDA-4 trial [46] (borderline reduction in heart failure), the intra-coronary route may be considered, but the intravenous route should remain the standard of care for administration of GPIs.

In the end, as determined by the ESC guidelines [3], routine use or upstream use of GPI is not included in the guidelines anymore. Using GPI as bailout therapy in the event of angiographic evidence of a large thrombus, slow flow/no-reflow phenomenon or other thrombotic complications is recommendable, although this strategy has not been tested in randomised trials.

#### Antithrombotic therapy after STEMI

##### Vorapaxar

Protease-activated receptors (PARs) are a family of guanine nucleotide-binding proteins- (G proteins-) coupled receptors, and PAR1 and PAR4 are expressed on human platelets. The only PAR1 antagonist that has completed phase III clinical investigations and is available for clinical use is vorapaxar. Vorapaxar is a synthetic tricyclic 3‑phenylpyridine that is an analogue of himbacine, and after oral administration, vorapaxar is rapidly absorbed with high bioavailability and a long half-life [47].

The TRACER trial was conducted in patients with NSTEMI and did not show a favourable balance between efficacy and bleeding with vorapaxar in acute management [48].

The TRA 2°P-TIMI 50 trial [49] was a secondary prevention trial designed to investigate the efficacy and safety of vorapaxar in the reduction of atherothrombotic events in patients with established atherosclerosis receiving aspirin and/or clopidogrel. Patients (*n* = 26,449) were randomly assigned to vorapaxar 2.5 mg daily or placebo. After a median follow-up of 30 months, vorapaxar significantly reduced the primary endpoint (death from cardiovascular causes, myocardial infarction, or stroke) by 13% compared with placebo, driven by a 17% reduction in the rate of myocardial infarction, at the expense of a significant increase in moderate or severe bleeding and a twofold increase in intracranial bleeding. Particularly in patients with previous STEMI (*n* = 9,248), vorapaxar led to a significant 27% reduction in the risk of cardiovascular death, myocardial infarction or stroke. The efficacy and safety of vorapaxar in combination with prasugrel and ticagrelor has not been tested. Future studies with different antiplatelet combinations, duration and doses are needed to clarify if vorapaxar has a role in the treatment of patients with STEMI. Vorapaxar (2.08 mg once daily maintenance dose) is currently approved by the FDA and EMA for the reduction of thrombotic events in patients with a history of myocardial infarction or peripheral arterial disease (Tab. 3).

##### Rivaroxaban

Rivaroxaban is a factor Xa inhibitor which does not require an antithrombin cofactor for its activity. In the setting after acute coronary syndrome (the ATLAS ACS-2 trial) 2.5/5 mg rivaroxaban twice daily combined with acetylsalicylic acid or acetylsalicylic acid plus clopidogrel demonstrated a statistically significant reduction of death from cardiovascular causes, myocardial infarction or stroke compared with placebo in patients after acute coronary syndrome [50]. However, there was also a reduction in all-cause and cardiovascular mortality, with an increased risk of major bleeding and intracranial bleeding but not of fatal bleeding. More recently, the GEMINI-ACS 1 trial, a phase 2 trial, [51] showed that a dual pathway antithrombotic therapy approach combining low-dose rivaroxaban (2.5 mg twice daily) with a P2Y12 inhibitor in the treatment of patients with acute coronary syndrome had a similar risk of clinically significant bleeding as aspirin and a P2Y12 inhibitor.

In patients with atrial fibrillation undergoing PCI, rivaroxaban may be considered as a therapeutic option as recently published in the PIONEER-AF trial [52]. Furthermore, in patients with stable angina, those assigned to rivaroxaban (2.5 mg twice daily) plus aspirin had better cardiovascular outcomes and more major bleeding events than those assigned to aspirin alone [53]. In patients with a low bleeding risk who receive aspirin and clopidogrel, low-dose rivaroxaban (2.5 mg twice daily) may be considered (Class 2b; LOE: B for both) as determined by the ESC guidelines (Tab. 3; [3]).

## Discussion

**Fig. 3:**
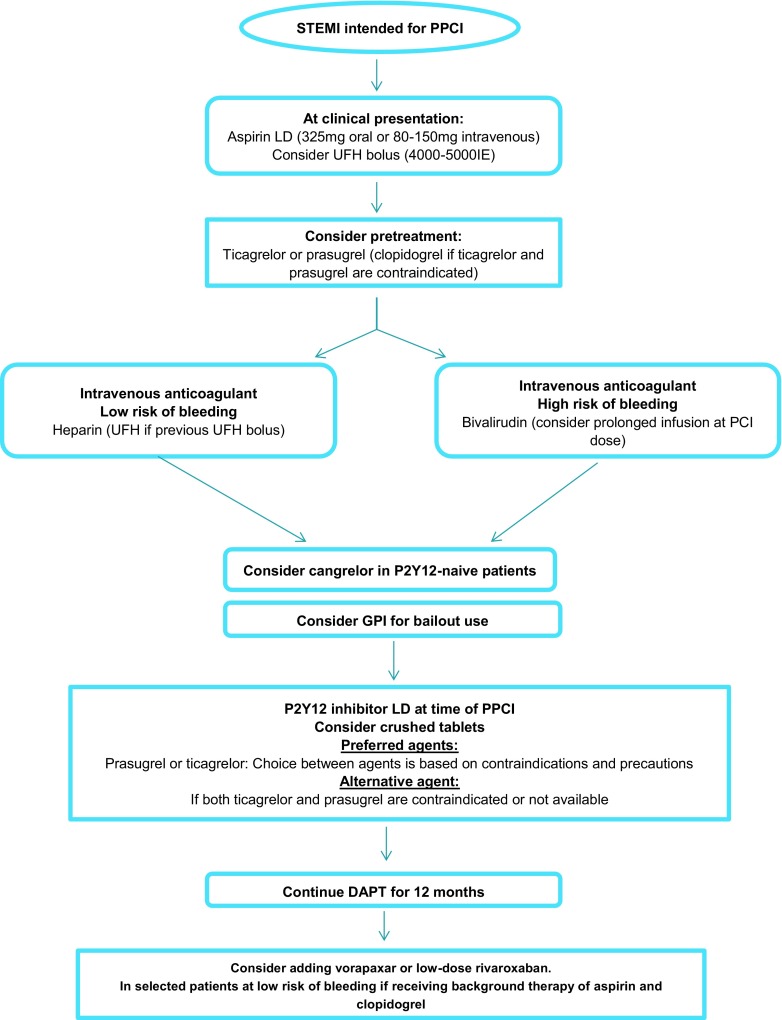
Proposed algorithms for the choice of antithrombotic therapy in STEMI patients undergoing primary PCI. *STEMI* ST-elevation myocardial infarction, *PPCI* primary percutaneous coronary intervention, *UFH* unfractionated heparin, *PCI* percutaneous coronary intervention, *LD* low dose, *DAPT* dual antiplatelet therapy, *GPI* glycoprotein IIb/IIIa inhibitor

The rationale for use of oral antithrombotic agents in the therapy of STEMI patients is modulating the effects of thrombin on both coagulation cascade and platelet aggregation, as well as lowering thrombotic complications, without increasing serious bleeding (Fig. 3). In patients with a low bleeding risk, the use of heparin, with or without GPI, as an anticoagulant seems to be cost-effective and has been associated with better clinical outcome. For patients not receiving upstream unfractionated heparin, enoxaparin can be considered as an alternative anticoagulant. In patients with a high bleeding risk bivalirudin should be considered. We need a strategy to reduce the risk of acute stent thrombosis if bivalirudin is used, such as an initial bolus of unfractionated heparin or prolonged infusion at PCI for up to 4 h after reperfusion.

With regard to antiplatelet therapy in STEMI, the recommend first-line P2Y12 inhibitors in the setting of primary PCI are prasugrel and ticagrelor [1–4]. Pre-treatment with P2Y12 inhibitors in STEMI has been tested in the ATLANTIC trial [27] and showed a significant reduction in the rate of acute stent thrombosis with no difference in major bleeding. However, pre-PCI markers of coronary reperfusion did not improve with pre-hospital use of ticagrelor. Nevertheless, in our opinion a loading dose of aspirin and potent P2Y12 inhibitors (ticagrelor or prasugrel) should be given as early as possible (upstream strategy) in STEMI patients. Maybe it is better to give the potent P2Y12 inhibitors crushed [54, 55] as it is effective and safe; pre-hospital feasibility will be tested in the Netherlands in the near future (ONTIME‑3 trial, clinicaltrials.gov nr NCT03400267). Clopidogrel use is reserved for patients when prasugrel or ticagrelor is contraindicated. However, there are several clinical conditions commonly associated with an inability to achieve adequate platelet inhibition with oral use of P2Y12 receptor inhibition, for example inability to swallow, nausea, shock and intubation.

For patients with these clinical conditions cangrelor is an option. It showed an enhanced platelet inhibition when administered in addition to prasugrel or ticagrelor therapy, but the clinical benefit of its use in addition to these agents should be tested in a pre-hospital STEMI trial.

Especially in high-risk STEMI patients, early GPI use may be considered as upstream therapy. However, there is no definitive answer regarding the current role of routine upstream use of GPI in primary PCI in the era of potent dual antiplatelet therapy, particularly when ticagrelor or prasugrel is used. The peri-procedural administration of GPI may be based on thrombus burden or in case with impaired haemodynamic conditions.

## Conclusions

Selecting the right antiplatelet and antithrombotic agents is of paramount importance in the treatment of STEMI patients undergoing primary PCI. New agents allow a reduction in rates of clinical events, including mortality, but this benefit may be reduced by the higher bleeding risk in some patients. Therefore, adjunctive pharmacotherapy should be tailored to the individual patient, based on assessment of ischaemic and bleeding risk. In this approach we decide on the optimal agent but also on the timing (pre-hospital, in catheterisation laboratory before angiography, or in catheterisation laboratory after angiography) and the means of administration (intravenous, intracoronary administration).
